# Molecular Cloning and Expression Analysis of Thyrotropin-Releasing Hormone, and Its Possible Role in Gonadal Differentiation in Rice Field eel *Monopterus albus*

**DOI:** 10.3390/ani12131691

**Published:** 2022-06-30

**Authors:** Ke Feng, Jialin Su, Zhengli Wu, Shengqi Su, Weizhi Yao

**Affiliations:** Key Laboratory of Freshwater Fish Reproduction and Development (Ministry of Education), College of Fisheries, Southwest University, Chongqing 400715, China; fengke163@swu.edu.cn (K.F.); suejialin@163.com (J.S.); zh20140202@swu.edu.cn (Z.W.); sushengqi@swu.edu.cn (S.S.)

**Keywords:** full-length cDNA, genomic DNA, qRT-PCR, sex hormone, maturation, reproductive physiology

## Abstract

**Simple Summary:**

Thyrotropin-releasing hormone (TRH) is an important upstream regulator in the hypothalamus-pituitary-thyroid (HPT) axis in mammals. In this study, we isolated and characterized *trh* gene from a protogynous hermaphrodite fish rice field eel *Monopterus albus*. TRH had no significant effect on serum thyroid hormone levels in rice field eel. However, we found that TRH was involved in the regulation gonadal differentiation-related gene expression and serum sex steroid hormone secretion. Our results indicated that TRH may play a novel role in gonadal differentiation in rice field eel.

**Abstract:**

Rice field eel (*Monopterus albus*), a protogynous hermaphrodite fish, is a good model for the research of sex determination and gonadal differentiation in teleosts. In this study, we cloned the full-length cDNA sequence of *trh*, which encoded a predicted protein with 270 amino acids. *Trh* mainly expressed in the brain, followed by the ovary, testis, muscle and pituitary, and had low levels in other peripheral tissues. During natural sex reversal, *trh* mRNA expression levels exhibited a significant increase at the late intersexual stage in the hypothalamus. In the gonad, *trh* mRNA expression levels showed a trend of increase followed by decrease, and only increased significantly at the middle intersexual stage. No matter static incubation or intraperitoneal (IP) injection, TRH had no significant effect on *trh* and *thyroid-stimulating hormone β* *subunit* (*tshβ*) mRNA expression levels, and serum T3, T4 and TRH release. After static incubation of ovarian fragments by TRH, the expression of *gonadal soma derived factor* (*gsdf*) was up-regulated significantly at both the doses of 10 and 100 nM. IP injection of TRH stimulated the expression of *gsdf*, and inhibited the expression of ovarian aromatase gene (*cyp19a1a*), accompanied by the increase of serum 11-KT levels. The results indicated that TRH may play a novel role in gonadal differentiation by the regulation of gonadal differentiation-related gene expression and sex steroid hormone secretion in rice field eel.

## 1. Introduction

Thyrotropin-releasing hormone (TRH) is a multifunctional hypophysiotropic factor in the hypothalamic-pituitary-thyroid (HPT) axis, which stimulates the release of pituitary hormones, and also plays roles in reproduction, feeding and energy balance in vertebrates [1,2,3]. TRH was first discovered from the hypothalamus of porcine and ovine, and was named for its role in the stimulation of thyrotropin (TSH) secretion in the pituitary [1,4,5]. Mature TRH is a kind of tripeptide (pGln-His-Pro-NH2), which is cleaved from a large precursor protein, preproTRH [6].

It is known that TRH stimulates the synthesis and release of TSH in the pituitary, and then promotes thyroid hormones (THs) synthesis and release in the thyroid gland in mammals [7]. In mouse, knock out of *trh* gene resulted in tertiary hypothyroidism and mild hyperglycemia, with a significant decrease of TH levels [8]. The deficiency of TRH receptor 1 (TRHR1) led to central hypothyroidism and mild hyperglycemia, accompanied by the increase of anxiety and depression levels in mouse [9]. However, the role of TRH in the HPT axis is species-specific in teleosts. TRH exhibited a positive stimulation on TSH in the pituitary in rainbow trout [10], bighead carp [11] and Japanese eel [12], while had no significant effect in tilapia [13], common carp [14] and coho salmon [15].

In teleosts, the *trh* gene has been identified in common carp, Senegalese sole, winter flounder, gilthead seabream, and so on [2,16,17,18]. As a hypothalamic releasing factor, TRH shows robust distribution in the brain, and is also detected in other peripheral tissues, especially in the gonad. It has found that *trh* mRNA exhibited different expression levels between ovary and testis in gilthead seabream [18]. The expression of *trhr* could also be detected in the gonad in the porcine and medaka [19,20]. Recently, there is an emerging concept of brain sex differentiation in teleosts, which means the sexual dimorphic expression of some neuroendocrine factors in the brain, and may be related to gonadal sex differentiation [21,22]. Gonadotropin-releasing hormone (GnRH) showed different expression between male and female brain, and played a pivotal role in gonadal differentiation in teleosts [21]. Just as GnRH, TRH is also an upstream neuroendocrine factor in the brain, but it is still unknown whether TRH is involved in this process by neuroendocrine or paracrine system.

Rice field eel, *Monopterus albus*, is an economically freshwater fish in China, which is considered to be the Synbranchidae and Synbranchiformes. In 2020, the total production of rice field eel reached more than 300,000 tonnes in China [23]. It is a typical protogynous hermaphrodite fish, which has a characteristic of natural sex reversal from functional females to males [24]. It has become a good model for the research on sex determination and gonadal differentiation in vertebrates [25]. So far, the regulator mechanism of natural sex reversal remains limited. Most studies mainly focus on sex differentiation-related genes in the gonad, such as *gonadal soma derived factor* (*gsdf*), *anti-Müllerian hormone* (*amh*), *SRY-related high-mobility group box 9* (*sox9*), *doublesex- and mab-3-related transcription factor-1* (*dmrt1*), *forkhead protein L2* (*foxl2*), and so on [26,27]. In this present study, we obtained the full-length cDNA and genomic sequences of *trh* at first, and detected *trh* mRNA distribution in various tissues in rice field eel. Then, we examined *trh* mRNA expression in hypothalamus and gonad at different developmental stages during sex reversal. We also evaluated the function of TRH in the HPT axis and the possible role in gonadal differentiation by in vitro and in vivo experiments in female rice field eel.

## 2. Materials and Methods

### 2.1. Experimental Fish and Collection of Samples

Wild rice field eels used in this study were obtained from a local aquatic product market in Chongqing, China. Fish was maintained in an indoor plastic aquarium for two weeks at room temperature under natural photoperiod at the aquaculture base of Southwest University. During the short period of temporary rearing, fish was fed with earthworm. Before sampling, all the fish were anesthetized with tricaine methanesulfonate (MS-222; Sigma, St. Louis, MO, USA). All experiments were conformed to the guidance of the Institutional Animal Care and Use Committees (IACUC) of Southwest University, Chongqing, China (Approval No: IACUC-20190823-01).

In order to obtained the cDNA sequence of *trh*, total RNA was extracted from hypothalamus. To clone *trh* genomic sequence, tail tissue was isolated and stored at 95% ethanol for DNA isolation. For tissue distribution detection, 13 tissues were isolated from five female fish with a mean body length (BL) of 33.04 ± 1.22 cm, including hypothalamus, pituitary, medulla oblongata, cerebellum, mesencephalon, telencephalon, muscle, heart, intestine, liver, spleen, kidney and ovary. Testis was isolated from another five male fish (BL = 40.60 ± 1.12 cm). The tissues were quickly collected, frozen immediately in liquid nitrogen, and transferred to the refrigerator at −80 °C until RNA extraction. After the conformation of phenotypic sex by histological observation of gonads under a light microscope, we collected the samples of gonad, hypothalamus and serum at different developmental stages during sex reversal. Five sexual phases were chosen for analysis, including ovary, ovotestis I, ovotestis II, ovotestis III and testis, according to the previous classification [28]. Five fish were sampled at each developmental stage.

### 2.2. RNA Isolation and cDNA Synthesis

Total RNA was isolated from various tissues using RNAiso Plus kit (Takara, Beijing, China). The purity and concentration were determined using a BioDrop (BioDrop, Cambridge, UK) spectrophotometer. The integrity was estimated with agarose gel electrophoresis. Based on the manufacturer’s protocol, 1 μg total RNA was taken as the template for cDNA synthesis using PrimeScript^TM^ RT reagent Kit with gDNA Eraser (Takara, Beijing, China).

### 2.3. cDNA and Genomic Cloning of trh Gene

To obtained the partial cDNA sequence of *trh*, two pairs of primers (*trh*-F1/*trh*-R1, *trh*-F2/*trh*-R2) were designed according to the predicted *trh* sequence in the rice field eel genome. The 20 μL PCR mixture contained 10 μL *Premix Taq*^TM^ (*LA Taq*^TM^ version 2.0 *plus dye*) (Takara, Beijing, China), 0.6 μL each of forward and reverse primers (10 mM), 7.8 μL ddH_2_O and 1.0 μL cDNA. PCR protocol was as follows: initial denaturation for 1 min at 98 °C, followed by 35 cycles of 10 s at 98 °C, 30 s at 60 °C, and 1.5 min at 72 °C, and a final extension for 5 min at 72 °C. In order to generate the full-length cDNA sequence of *trh*, rapid amplification of cDNA ends (RACE) was performed according to the classical method [29].

To obtained the *trh* genomic sequence, DNA was isolated from fish tail using DNAiso reagent (Takara, Beijing, China) according to the standard protocol. Two pairs of primers (*trh*-F1/3’RACE outer, *trh*-Q-F1/*trh*-Q-R1) were used to amplify introns of *trh* gene, respectively. All the target PCR products were purified with a gel recovery kit (Dingguo, Beijing, China), and ligated into the pMD19-T vector (Takara, Beijing, China). After transformation into *Escherichia coli* DH5α competent cells (Takara, Beijing, China), the recombinant plasmid was confirmed by DNA sequencing of positive clones. In this study, all the primers were designed using the classical software of Primer Premier 5.0 (Premier Biosoft International, Palo Alto, CA, USA) (Table 1). Both the synthesis of primers and sequencing of positive products were performed by Sangon Biotech Company in Shanghai, China.

### 2.4. Bioinformatic Analysis of Sequences

The full-length cDNA sequence of *trh* was obtained by assemblage of the sequences of partial cDNA fragment, 3’RACE and 5’RACE. The prediction of open reading frame (ORF) was carried out using online tool on NCBI website (https://www.ncbi.nlm.nih.gov/orffinder/, accessed on 27 October 2021). The Splign program was used to distinguished exons and introns on NCBI website (https://www.ncbi.nlm.nih.gov/sutils/splign/splign.cgi?textpage=online&level=form, accessed on 27 October 2021) [30]. The prediction of signal peptide and basic properties of protein were performed using SinalP 5.0 server (https://services.healthtech.dtu.dk/service.php?SignalP-5.0, accessed on 27 October 2021) and the ProtParam web tool (https://web.expasy.org/protparam/, accessed on 27 October 2021), respectively [31]. Multiple alignment and similarity of amino acid sequences were determined with the Clustal X 2.0 software (Version 2.0, the Conway Institute UCD Dublin, Heidelberg, Germany) [32]. The construction of a phylogenetic tree based on the protein sequences was performed on the MEGA 7.0 software (Version 7.0, Tokyo Metropolitan University, Tokyo, Japan) using neighbor-joining (NJ) method [33].

### 2.5. Analysis of Quantitative Real-Time PCR (qRT-PCR)

The qRT-PCR was carried out on a BioRad CFX96^TM^ Real-Time PCR Detection System. The 20 μL PCR mixture included 10 μL TB Green^®^ *Premix Ex Taq*^TM^ II (Tli RNaseH Plus) (Takara, Beijing, China), 0.6 μL each of forward and reverse primers (10 mM), 6.8 μL ddH_2_O and 2 μL 5-fold diluted cDNA template. PCR protocol was as follows: heated to 95 °C for 30 s, followed by 40 cycles at 95 °C for 5 s, 60 °C for 30 s. Primer specificity was detected by a melt curve analysis from 65 °C to 95 °C. Three duplicates was set up for each template, and a negative control was contained on each PCR plate. The expression level of *elongation factor-1-α* (*ef1α*) was relatively stable, and was suitable as reference gene in different tissues and gonads of different developmental stages [28]. Therefore, the expression level of target gene was normalized to *ef1α*, and was compared using relative Ct method [34].

### 2.6. Static Incubation Experiments

TRH (protirelin acetate) with purity of 99% was obtained from Macklin Biochemical Technology Company in Shanghai, China. Based on the manufacturer’s instruction, it was dissolved in double distilled water and stored at −20 °C. Prior to use, the stock solution was diluted into three different concentrations (1, 10 and 100 nM) by phosphate-buffered saline (PBS, pH = 7.2). The concentrations of TRH used for static incubation were conformed based on the previous studies [11,35]. The hypothalamus, pituitary and ovary fragments of female eels were prepared based on previous studies [36,37,38]. After washed three times in PBS, the tissue was cut into small pieces with scissors. Hypothalamus and pituitary were pre-incubated for 1 h, while ovary for 18 h in M199 medium (Gibco, Thermo Fisher Scientific, Waltham, MA, USA) including penicillin and streptomycin at the final concentration of 100 U/mL. The incubation was carried out in a 5% carbon dioxide incubator at 25 °C with 100% humidity. After pre-incubation, the fragments were placed into 24-well plates (Corning, Amsterdam, The Netherland), and incubated with fresh culture medium containing different concentrations of TRH. Each treatment was performed in 3 well replicates. Hypothalamus from two fish was mixed in one well, and pituitary from three fish was mixed in one well, respectively. The ovary was cut into small fragments and divided evenly into 12 wells. The third stage of ovary [28] from four eels (BL = 36.48 ± 2.32 cm) was chosen for analysis, which was confirmed by observing paraffin section. The tissue fragments were sampled for total RNA isolation after 6 h of the incubation.

### 2.7. The Design of Intraperitoneal (IP) Injection Experiment

The IP injection experiment was performed according to our previous study [39]. Briefly, after acclimatization for two weeks, the female eels (BL = 37.12 ± 0.25, n = 20) in the third ovary stage were chosen for IP injection. The stock solution of TRH was diluted in fish physiological saline. The selection of injection doses (0.01, 0.1, 1, 10 and 100 ng/g body weight) was referred to previous study [39]. At the same time, an injection with only fish physiological saline was taken as the control. Six hours after IP injection, 1 mL blood was obtained for the measurement of serum hormones, and hypothalamus, pituitary and ovary were sampled for RNA isolation. Five eels were sampled at each dose.

### 2.8. Serum Hormones Assays

After standing for 2 h at room temperature, blood samples were transferred to refrigerator at 4 °C overnight. After centrifugation (4 °C, 5000 r/min, 20 min), serum was collected from the supernate and stored at −20 °C for further analysis. The concentrations of serum T3, T4, estradiol-17β (E2) and 11-ketotestosterone (11-KT) were determined using commercial enzyme-linked immunosorbent assay kits (Preferred Biotechnology, Shanghai, China) following the manufacturer’s protocol. The inter- and intra-assay coefficients of variation were 4.43% and 6.67% for T3, 3.13% and 2.35% for T4, 5.36% and 9.45% for TRH, 11.60% and 14.48% for E2, and 11.35% and 17.59% for 11-KT, respectively.

### 2.9. Data Analysis

All experimental values were expressed as mean ± standard error (SE). Statistical analysis was performed with Statistica version 10.0 software (Statsoft Inc., Tulsa, OK, USA). Prior to data analysis, the homogeneity of variance was tested using the Levene’s test. Then the data were subjected to one-way analysis of variance (ANOVA), and differences were determined by Tukey’s test. Significant differences were set at *p*< 0.05.

## 3. Results

### 3.1. Molecular Characterization of trh

**Figure 1:**
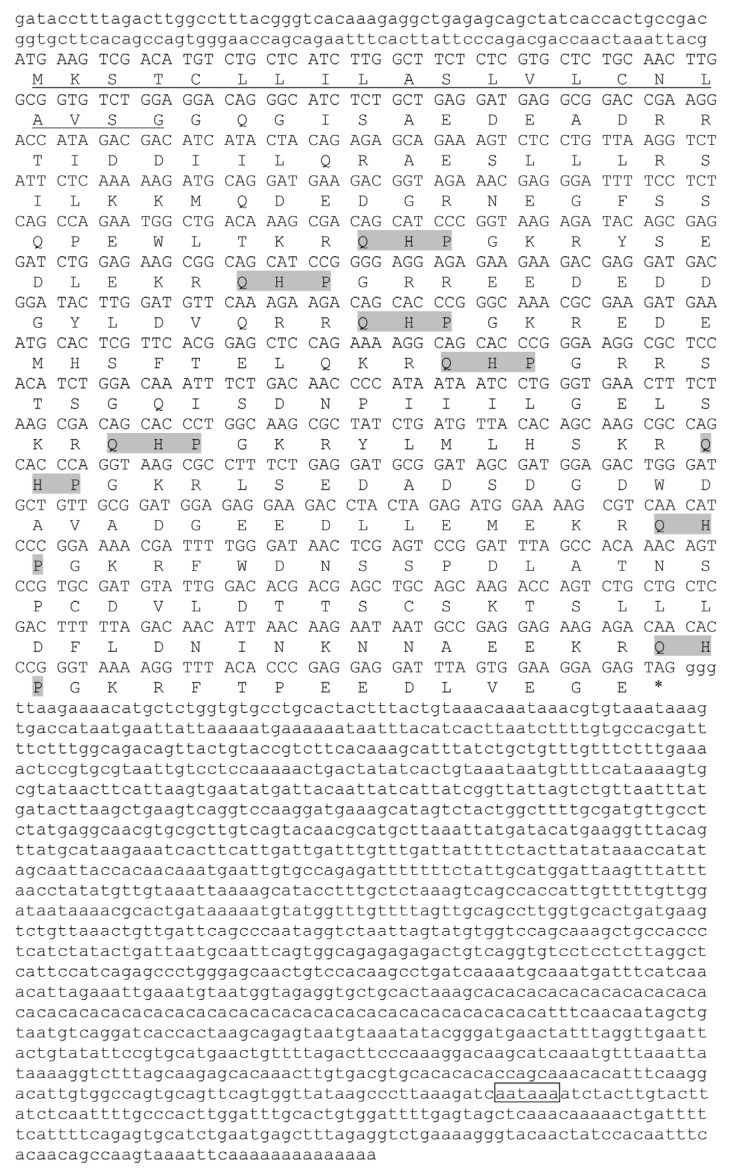
Nucleotide and predicted amino acid sequences of *trh*. 5′-, 3′-untranslated regions are exhibited in lowercase. Uppercase indicates the coding region. Uppercase below coding region indicates the predicted amino acids. Signal peptide is marked with an underscore. An asterisk (*) indicates the stop codon. The mature peptides (TRH) and putative polyadenylation signal are shaded and boxed, respectively.

The full-length *trh* cDNA sequence (GenBank accession number: OM324394) was 2410 bp in length (Figure 1). The ORF was 813 bp in length, which encoded a preproTRH with 270 amino acids (aa). The length of 5’ untranslated region (UTR) and 3’UTR was 130 bp and 1467 bp, respectively. The molecular formula of preproTRH was C_1315_H_2111_N_407_O_437_S_9_, and the relative molecular weight was 30,903.23 Da. The preproTRH was predicted to be unstable and hydrophilic, with an instability index of 72.66 and a grand average of hydropathicity (GRAVY) of −1.125, respectively. The preproTRH contained a 21 aa predicted signal peptide and eight copies of TRH progenitor sequence (Gln-His-Pro-Gly). The *trh* gene was 3327 bp in length, which contained three exons and two introns (GenBank accession number: OM324395). The examination of exon-intron boundary sequence suggested that they conformed to the typical GT-AG rule.

### 3.2. Sequence Alignment and Phylogenetic Tree Analysis

**Figure 2:**
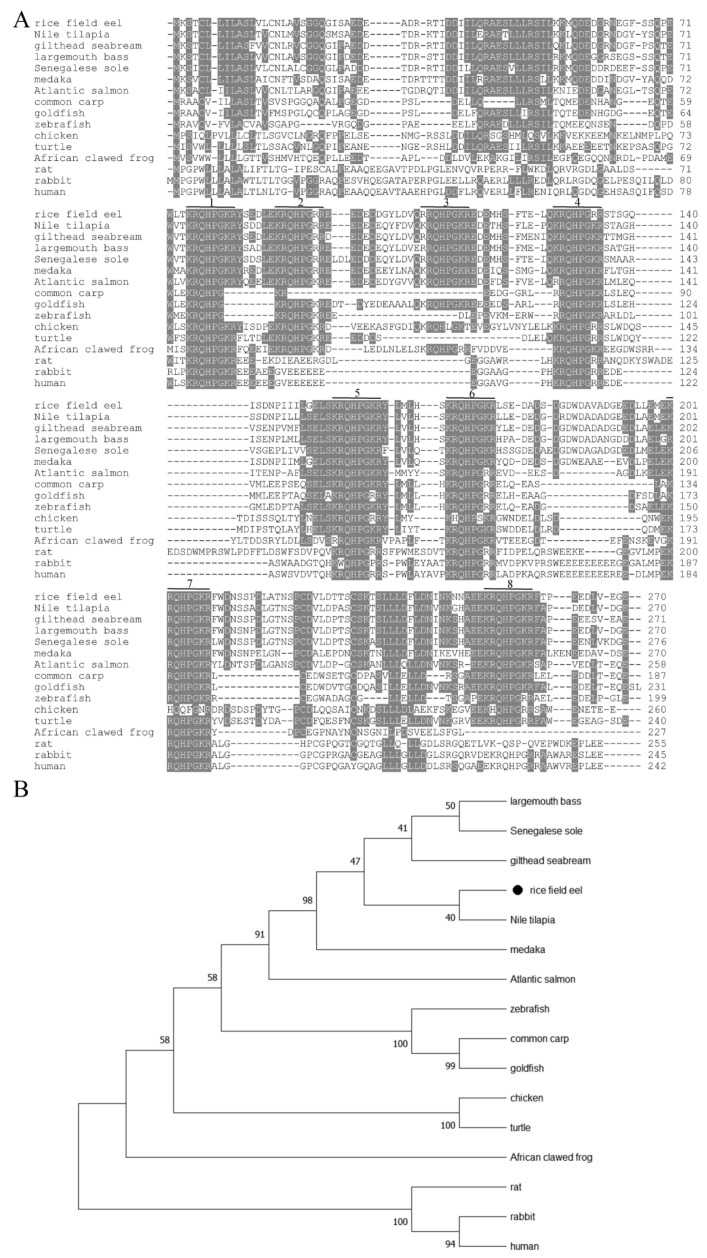
Bioinformatics analysis of preproTRH. (**A**) Sequence alignment of predicted preproTRH. TRH progenitor sequences are shaded and numbered. (**B**) Molecular phylogenetic tree of preproTRH. The numbers at tree nodes indicate the bootstrap values (%). “●” indicates rice field eel preproTRH. GenBank accession numbers of preproTRH sequences: largemouth bass, *Micropterus salmoides*, XP_038578202.1; Senegalese sole, *Solea senegalensis*, CBA18273.1; Nile tilapia, *Oreochromis niloticus*, XP_003439044.1; gilthead seabream, *Sparus aurata*, XP_030277807.1; medaka, *Oryzias latipes*, NP_001098204.1; Atlantic salmon, *Salmo salar*, NP_001134644.1; zebrafish, *Danio rerio*, NP_001012365.2; common carp, *Cyprinus carpio*, BAD83781.1; goldfish, *Carassius auratus*, BAD83782.1; chicken, *Gallus gallus*, NP_001025554.1; turtle, *Mauremys mutica*, XP_044881250.1; African clawed frog, *Xenopus laevis*, AAA49973.1; rat, *Rattus norvegicus*, NP_037178.1; rabbit, *Oryctolagus cuniculus*, XP_002713086.2; human, *Homo sapiens*, NP_009048.1.

A comparison of predicted amino acid sequence of TRH with that of other vertebrates revealed that TRH sequences were relatively conserved (Figure 2A). Eel TRH displayed the same high similarity of 83.3% to that of Nile tilapia, gilthead seabream and largemouth bass. It shared higher similarity (48.7–80.0%) with other teleost TRH, and had lower similarity with that of rabbit (37.3%) and rat (33.2%). A phylogenetic tree was generated based on the alignment of published vertebrate TRH amino acid sequences on NCBI website (Figure 2B). The results showed that rice field eel was grouped closed to Nile tilapia, gilthead seabream, Senegalese sole and largemouth bass in teleost. Teleost clustered into a big branch, and was separated from other vertebrates. Overall, the result of phylogenetic tree analysis of TRH sequences was agreement to the predicted pattern of species evolution.

### 3.3. The Expression Pattern of trh mRNA in Various Tissues and during Sex Reversal

**Figure 3:**
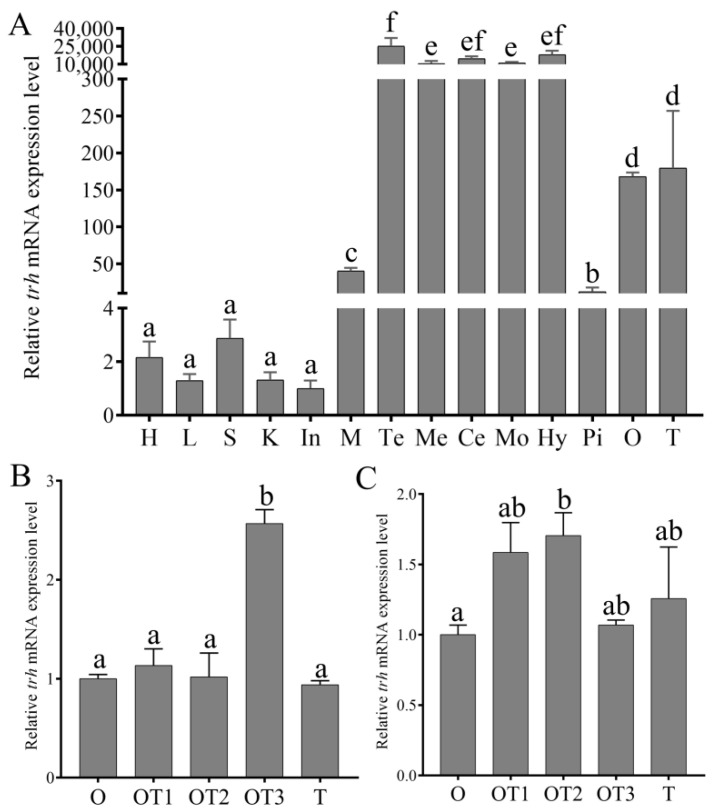
Relative expression levels of *trh* in various tissues (**A**), and different stages of hypothalamus (**B**) and gonad (**C**) during sex reversal (*n* = 5). Heart (H), liver (L), spleen (S), kindey (K), intestin (In), muscle (M), telencephalon (Te), mesencephalon (Me), cerebellum (Ce), medulla oblongata (Mo), hypothalamus (Hy), pituitary (Pi), ovary (O), testis (T), ovotestis I (OT1), ovotestis II (OT2), ovotestis III (OT3). Values with different superscript denote significantly difference (*p* < 0.05).

The *trh* mRNA could be found in all the detected tissues by qRT-PCR (Figure 3A, Table S1). It mainly expressed in the brain region, and had the highest expression level in the telencephalon, followed by the hypothalamus, cerebellum, medulla oblongata and mesencephalon. Moderate expression level was found in the ovary, testis, muscle and pituitary. Low expression level was detected in other peripheral tissues. In the hypothalamus, the expression level of *trh* only increased significantly at the stage of ovotestis III (Figure 3B). In the gonad, *trh* expression level showed a trend of increase followed by decrease, and only increased significantly at the stage of ovotestis II (Figure 3C).

### 3.4. Roles of TRH on the Regulation of HPT Axis In Vitro and In Vivo

**Figure 4:**
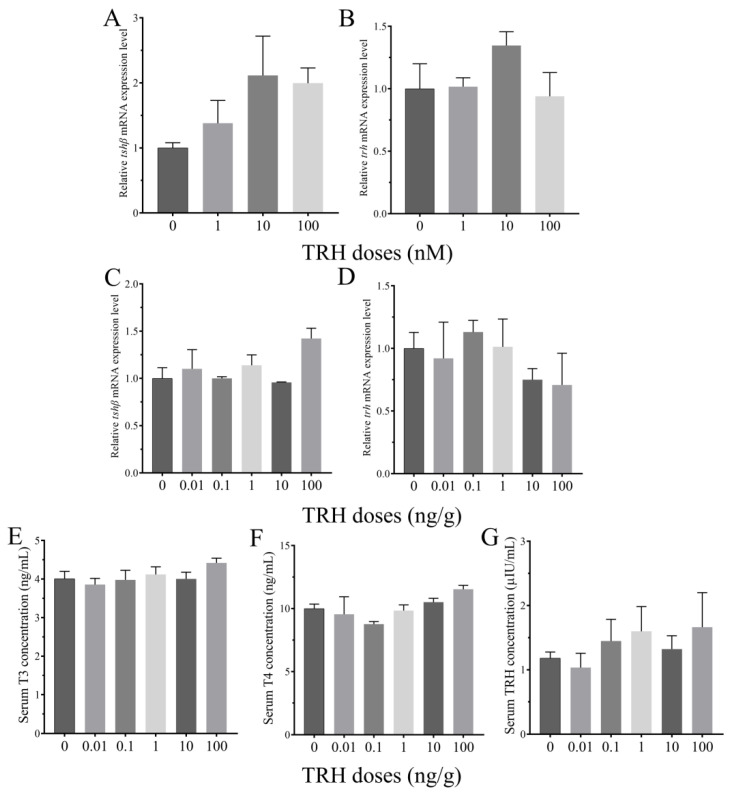
Effects of TRH on the regulation of HPT axis.(**A**,**B**) Relative expression levels of *tshβ* in the pituitary and *trh* in the hypothalamus after static incubation of TRH (*n* = 3). (**C**,**D**) Relative expression levels of *tshβ* in the pituitary and *trh* in the hypothalamus after IP injection of TRH (*n* = 5). (**E**–**G**) Serum T3, T4 and TRH levels after IP injection of TRH (*n* = 5).

Compared to the control group, *tshβ* mRNA expression level in the pituitary was up-regulated by the static incubation with different doses of TRH, but the difference was not significant (Figure 4A). Static incubation of TRH also had no significant change on *trh* mRNA expression in the hypothalamus (Figure 4B). IP injection with different doses of TRH had no significant effect on *tshβ* mRNA expression in the pituitary (Figure 4C). Compared to the control group, the expression level of *trh* was down-regulated at both the doses of 10 and 100 ng/g, but the difference was not significant (Figure 4D). Serum T3 concentration had no significant difference by IP injection of TRH (Figure 4E). Compared to the control group, TRH treatment resulted in a slight increase of serum T4 and TRH levels at the high doses, but the difference was not significant (Figure 4F,G).

### 3.5. In Vitro Effects of TRH on the Expression of gsdf, amh and cyp19a1a

**Figure 5:**
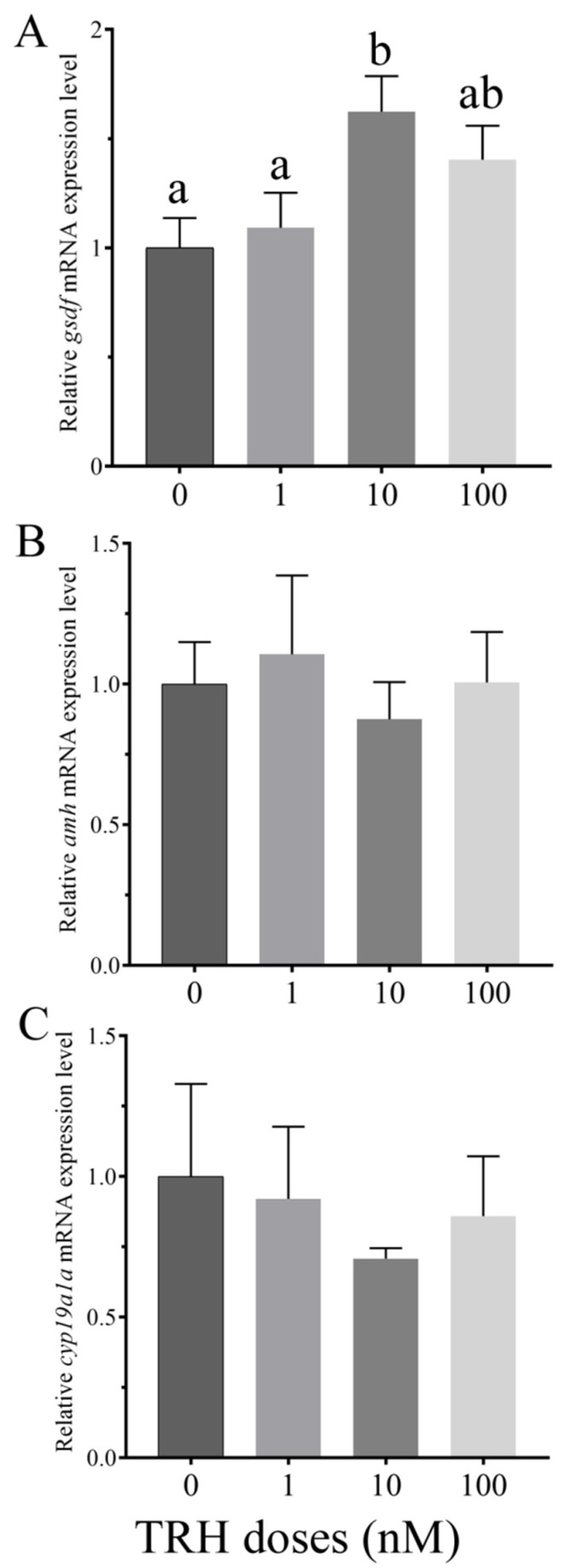
Relative expression levels of *gsdf* (**A**), *amh* (**B**) and *cyp19a1a* (**C**) in the third stage ovary of rice field eel after static incubation of TRH (*n* = 4). Values with different superscript denote significantly difference (*p* < 0.05).

In order to examine the role of TRH on gonadal differentiation, the expression of three important gonadal differentiation-related genes (*gsdf*, *amh* and *cyp19a1a*) was detected by static incubation of ovarian fragments with TRH. Compared to the control group, *gsdf* mRNA expression level had no significant increase at the dose of 1 nM, and was significantly higher at the dose of 10 nM, but the increase was no significant at the dose of 100 nM (Figure 5A). The expression of *amh* had no significant change at all the three different doses (Figure 5B). After static incubation of TRH, *cyp19a1a* mRNA expression had a slight decrease, but the difference was not significant (Figure 5C).

### 3.6. In Vivo Effects of TRH on the Expression of gsdf, amh and cyp19a1a

**Figure 6:**
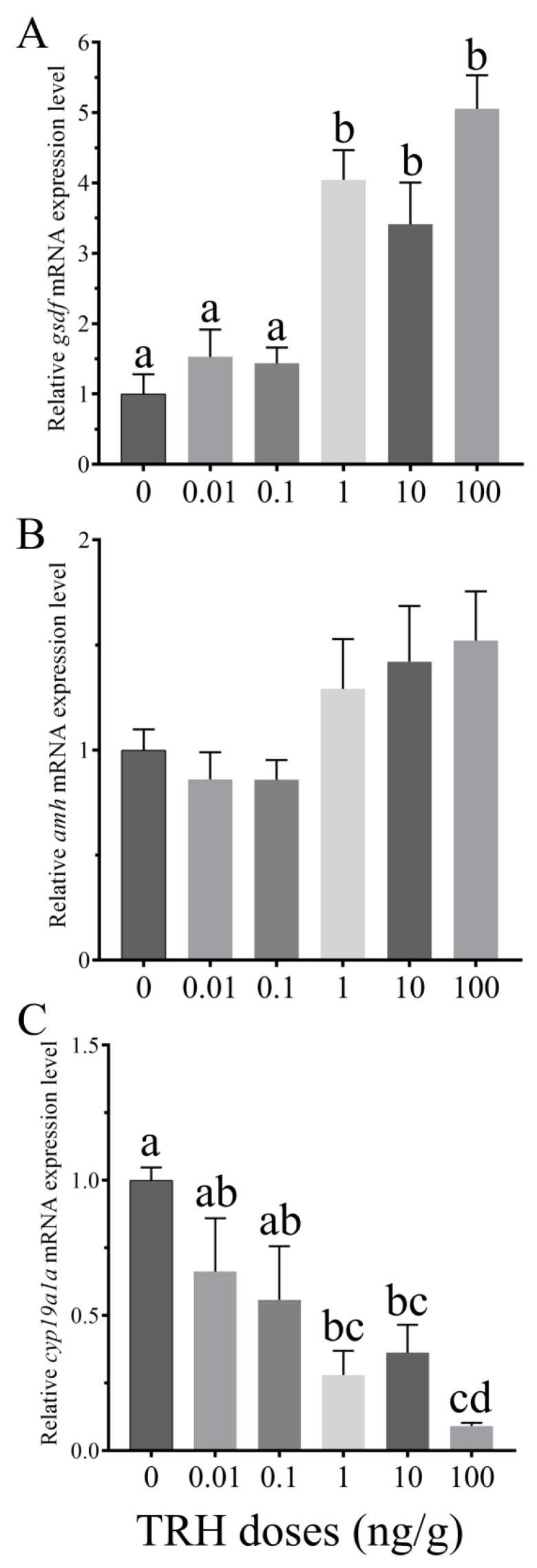
Relative expression levels of *gsdf* (**A**), *amh* (**B**) and *cyp19a1a* (**C**) in the third stage ovary of rice field eel after intraperitoneal injection of TRH (*n* = 5). Values with different superscript denote significantly difference (*p* < 0.05).

IP injection of TRH had no significant effect on the expression level of *gsdf* at lower doses of 0.01 and 0.1 ng/g, while significantly up-regulated the expression at the higher doses of 1, 10 and 100 ng/g(Figure 6A). Compared to the control group, *amh* increased slightly with the dose increase, but the difference was not significant (Figure 6B). TRH treatment resulted in a decrease trend of *cyp19a1a* mRNA expression level, and decreased significantly at doses of 1, 10 and 100 ng/g (Figure 6C).

### 3.7. Effect of TRH Injection on Serum E2 and 11-KT Levles

**Figure 7:**
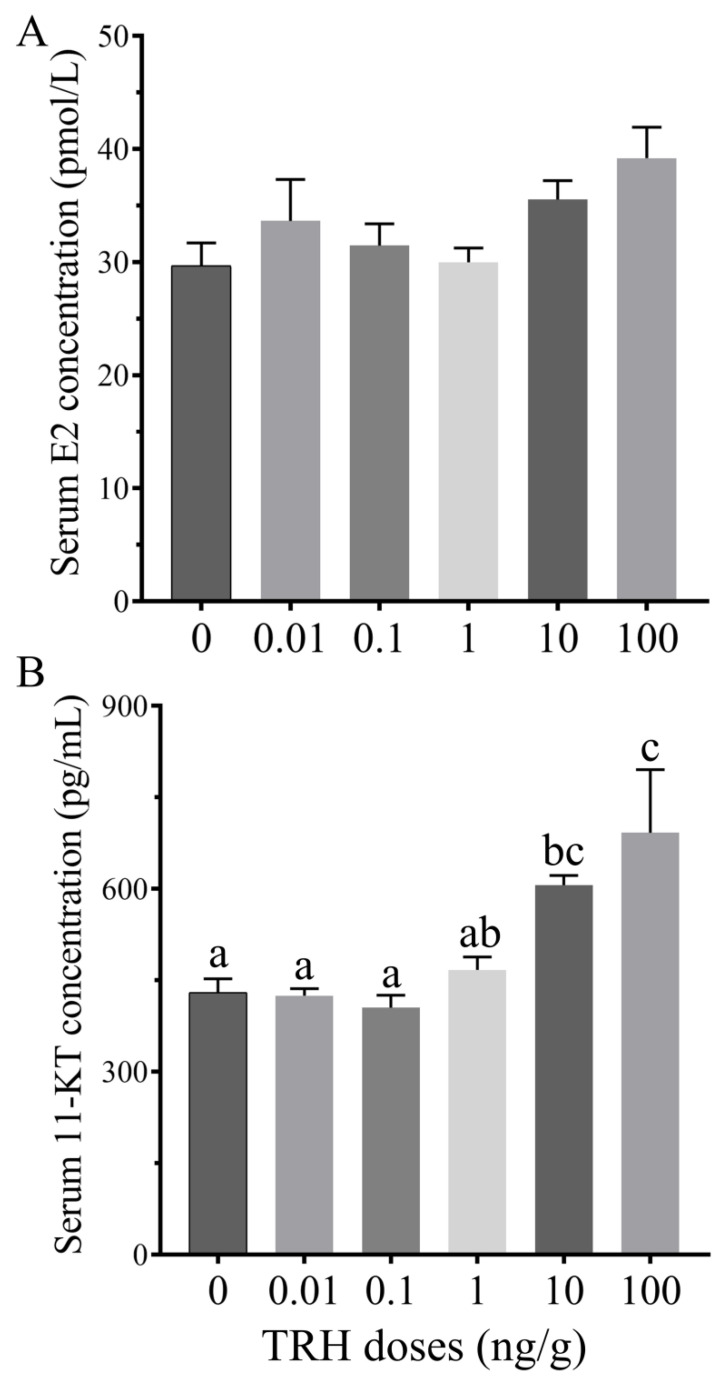
Effects of TRH injection on the levels of serum E2 (**A**) and 11-KT (**B**) (*n* = 5). Values with different superscript denote significantly difference (*p* < 0.05).

In order to evaluate the effect of TRH injection on estrogen and androgen production, we detected serum E2 and 11-KT levels. TRH stimulated a slight increase of serum E2 concentration, but the difference was not significant at all the five different doses (Figure 7A). After IP injection of TRH, serum 11-KT level did not show significant change at doses of 0.01, 0.1, and 1 ng/g, but increased significantly at doses of 10 and 100 ng/g (Figure 7B).

## 4. Discussion

In this study, we obtained the sequences of full-length cDNA and genomic DNA sequences of *trh* in rice field eel. Multiple sequence alignment of preproTRH indicated that TRH progenitor sequences had high identity, while the identity of other regions was low. Based on the known characteristic of prohormone convertases, only site (Lys-Arg or Arg-Arg) could be recognized and proteolytically cleaved [40]. Previous studies suggested that preproTRH contains several copies of TRH progenitor sequence, but the number is different among vertebrates from four to eight [2,6,16,17,18,39,41,42,43,44]. Our results suggested that rice field eel preproTRH contained eight copies of progenitor sequence, which was the same as that in most teleosts. The reason of copy number difference may be due to gene duplication, deletion or mutation during evolution [6,17]. In frog, two or more mRNAs of *trh* could be detected by northern blot analysis, and at least two similar *trh* genes may exist in the genome [43]. In rice field eel, only one copy of *trh* gene was obtained. The composition of *trh* gene in rice field eel was the same as that in medaka, chicken, rat and human [40,41,42,44]. The result indicated that the genomic structure of *trh* is relatively conserved during evolution in vertebrates.

As a hypothalamic releasing factor, *trh* was mainly expressed in the brain in vertebrates. Besides of the brain, *trh* was also present in the skin and eyes by northern blot analysis in frog [45]. In catfish, TRH could be detected in the brain and pituitary at mRNA and protein levels by the detection of qRT-PCR and immunofluorescence [46]. More and more researches suggested that *trh* was wildly expressed in other peripheral tissues in teleosts, such as winter flounder, gilthead seabream and Senegalese sole [2,17,18]. In our study, *trh* exhibited high expression level in the brain regions, moderate in the ovary and testis, and low in other peripheral tissues. The expression of *trh* could not be detected in peripheral tissues in some teleosts, probably due to the fact that the detection methods was less sensitive or species-specific differences. It was worth noting that moderate expression level of *trh* was found in the gonad. The expression level of *trh* in the hypothalamus and gonad showed different trend during sex reversal. So far, no much research focus on the expression difference of *trh* during sex reversal in teleosts. In gilthead seabream, a protandrous hermaphrodite teleost, the expression level of *trh* was higher in the testis than that in the ovary [18]. In medaka, two *trhr* subtypes (*trhr2* and *trhr3*) were expressed both in the ovary and testis by RT-PCR detection [20]. In our study, four *trhr* subtypes of were found in rice field eel, and only *trhr2* could be detected in ovary and testis (Figure S1). The tissue expression pattern indicated that TRH may exhibit multiple functions in different tissue, and may play roles in gonadal differentiation during sex reversal in rice field eel.

Unlike mammals, the role of TRH in the HPT axis is still uncertain in teleosts. In some TRH treatment experiments, TRH up-regulated the expression of *tshβ* and stimulated the synthesis and secretion of TH [11,12,47], but some other showed TRH had no significant or inhibitory effect on thyroid activity by pituitary-thyroid axis [14,15,48]. In cultured pituitary cells, TRH treatment had no significant effect on TSH secretion, but CRH caused a significant increase at the same concentration, and CRH injection also showed stimulatory effect in coho salmon [15,47]. There existed the adjacent localization between CRH-immunoreactive fibers and TSH-immunoreactive cells in the pituitary of juvenile chinook salmon, providing the possible evidence on the regulation of CRH in TSH release [49]. In this study, no matter in vitro or in vivo, TRH showed no significant change on the expression of *trh* and *tshβ*, and the release of serum T3, T4, and TRH. Our results indicated that TRH may not regulate the synthesis and release of TH in rice field eel. However, it needs further study to investigate the possible dose or time effects of TRH in the HPT axis. What’s more, further studies should be performed to explore whether CRH acts as the TSH-releasing factor in rice field eel.

The process of sex reversal is complex, which may be the combined effects of genetic and environmental factors. Some neuroendocrine factors showed sexually dimorphic expression at the level of brain, indicating they may also play roles in the regulation of gonadal sex differentiation in teleosts [21]. Previous studies demonstrated that THs had roles in sex differentiation, testicular development and spermatogenesis by the regulation of sex-related gene expression in teleosts [50]. Although TRH has no effect on the regulation of HPT axis in some teleosts, it is not clear whether TRH has a novel role in gonadal differentiation. GSDF is a member of transforming growth factor-beta (TGF-β) superfamily, which is essential for testicular differentiation and sex reversal [51]. AMH is also a member of TGF-β superfamily, which is involved in gonadal development by the inhibition of germ cell proliferation and differentiation [52]. Gonadal aromatase, encoded by *cyp19a1a*, is mainly distributed in the ovary and is crucial for ovarian differentiation in teleosts [53]. During female-to-male sex reversal, the expression levels of *gsdf* and *amh* showed continuous increase, while *cyp19a1a* decreased in the gonad in rice field eel [54,55,56]. In genetically female Japanese flounder, the masculinization was induced by the exogenous androgen and high temperature, accompanied by the increase of *gsdf* and *amh* expression, and decrease of *cyp19a1a* expression [57]. In our study, static incubation of ovary by TRH stimulated the expression of *gsdf*. TRH injection resulted in the upregulation of *gsdf* and *amh* expression, and downregulation of *cyp19a1a* expression. At the same time, TRH injection resulted in the change of serum sex steroid hormones, with a significant increase of serum 11-KT level. The results indicated that TRH may be involved in gonadal differentiation by the regulation of sex-related gene expression and sex steroid hormones. Since TRH had no significant effect on serum TRH levels at different IP injection doses, and there was *trhr* gene (*trhr2*) expressed in the gonad, we speculated that TRH played a direct role in gonadal differentiation. However, the regulatory mechanism of TRH in this process still needs more in-depth studies.

## 5. Conclusions

In summary, for the first time, we identified the full length cDNA and genomic sequences of *trh*, and examined the distribution in various tissues in rice field eel. During sex reversal, *trh* mRNA expression level changed in the hypothalamus and gonad at different developmental stages, indicating it may play roles in the process of gonadal differentiation. Based on the results of static incubation and IP injection of TRH, we discovered that it may not be involved in the regulation of TH secretion through HPT axis. In particular, we firstly demonstrated that TRH may have a novel role in gonadal differentiation by the regulation of gonadal differentiation-related gene expression and serum sex steroid secretion in rice field eel. The results provide new data to biological function research of TRH in teleosts.

## Figures and Tables

**Table 1:** Primers used in this study.

Name	Primers (5′→3′)	Use
*trh*-F1	TTCACAGCCAGTGGGAACC	cDNA fragment cloning
*trh*-R1	ATAGAGGCAACATCGCAAAAG	
*trh*-F2	TCCGTGCGTAATTGTCCTCC	
*trh*-R2	CTGGCCACAATGTCCTTGAA	
oligo-dT adaptor	GACTCGAGTCGACATCGA(T)17	adaptor primers for RACE
adaptor	GACTCGAGTCGACATCGA	
3′RACE outer	GCAACTGTCCACAAGCCTGAT	3′RACE cloning
3′RACE inner	TCCCAAAGGACAAGCATCAA	
5′RACE outer	CGTCTATGGTCCTTCGGTCC	5′RACE cloning
5′RACE inner	GCACGAGAGAAGCCAAGATG	
*trh*-Q-F1	CGGACCGAAGGACCATAGAC	real-time PCR
*trh*-Q-R1	GATGCTGTCGCTTTGTCAGC	
*tshβ*-Q-F1	GCTGTTCCCACGTGTTTACC	
*tshβ*-Q-R1	GGAAGCGTGGGCCAAGAATA	
*gsdf*-Q-F1	ATACCCACATCCTGCACAACG	
*gsdf*-Q-R1	TGAAGATCGCATTTGGAGAG	
*amh*-Q-F1	TGCTCCCGACGTTTGAAG	
*amh*-Q-R1	GAATGTTGCCCTCTGATGC	
*cyp19a1a*-Q-F1	TACTCAGCAGGTCATCAGCG	
*cyp19a1a*-Q-R1	TCTCATTGACAGGTACACCA	
*ef1a*-Q-F1	CGCTGCTGTTTCCTTCGTCC	
*ef1a*-Q-R1	TTGCGTTCAATCTTCCATCCC	

## Data Availability

All datasets generated or analyzed during this study are included in the published article.

## Supplementary Materials

The following supporting information can be downloaded at: https://www.mdpi.com/article/10.3390/ani12131691/s1. Figure S1. Distribution of *trhr* in rice field eel tissues. Table S1. The relative expression data of *trh* in various tissues. Table S2. Primers used for the detection of tissue distribution of *trhr* by RT-PCR.

## References

[1] Galas L., Raoult E., Tonon M.C., Okada R., Jenks B.G., Castano J.P., Kikuyama S., Malagon M., Roubos E.W., Vaudry H. (2009). TRH acts as a multifunctional hypophysiotropic factor in vertebrates. Gen. Comp. Endocrinol..

[2] Buckley C., MacDonald E.E., Tuziak S.M., Volkoff H. (2010). Molecular cloning and characterization of two putative appetite regulators in winter flounder (*Pleuronectes americanus*): Preprothyrotropin-releasing hormone (TRH) and preproorexin (OX). Peptides.

[3] Deal C.K., Volkoff H. (2021). Response of the thyroid axis and appetite-regulating peptides to fasting and overfeeding in goldfish (*Carassius auratus*). Mol. Cell. Endocrinol..

[4] Burgus R., Dunn T.F., Desiderio D., Ward D.N., Vale W., Guillemin R. (1970). Characterization of ovine hypothalamic hypophysiotropic TSH-releasing factor. Nature.

[5] Nair R.M., Barrett J.F., Bowers C.Y., Schally A.V. (1970). Structure of porcine thyrotropin releasing hormone. Biochemistry.

[6] Wallis M. (2010). Molecular evolution of the thyrotrophin-releasing hormone precursor in vertebrates: Insights from comparative genomics. J. Neuroendocrinol..

[7] Chiamolera M.I., Wondisford F.E. (2009). Thyrotropin-releasing hormone and the thyroid hormone feedback mechanism. Endocrinology.

[8] Nakajima Y., Yamada M., Taguchi R., Shibusawa N., Ozawa A., Tomaru T., Hashimoto K., Saito T., Tsuchiya T., Okada S. (2012). NR4A1 (Nur77) mediates thyrotropin-releasing hormone-induced stimulation of transcription of the thyrotropin β gene: Analysis of TRH knockout mice. PLoS ONE.

[9] Zeng H.K., Schimpf B.A., Rohde A.D., Pavlova M.N., Gragerov A., Bergmann J.E. (2007). Thyrotropin-releasing hormone receptor 1-deficient mice display increased depression and anxiety-like behavior. Mol. Endocrinol..

[10] Eales J.G., Himick B.A. (1988). The effects of TRH on plasma thyroid hormone levels of rainbow trout (*Salmo gairdneri*) and arctic charr (*Salvelinus alpinus*). Gen. Comp. Endocrinol..

[11] Chatterjee A., Hsieh Y.L., Yu J.Y.L. (2001). Molecular cloning of cDNA encoding thyroid stimulating hormone β subunit of bighead carp *Aristichthys nobilis* and regulation of its gene expression. Mol. Cell. Endocrinol..

[12] Han Y.S., Liao I.C., Tzeng W.N., Yu J.Y.L. (2004). Cloning of the cDNA for thyroid stimulating hormone beta subunit and changes in activity of the pituitary-thyroid axis during silvering of the Japanese eel, *Anguilla japonica*. J. Mol. Endocrinol..

[13] Melamed P., Eliahu N., Levavi-Sivan B., Ofir M., Farchi-Pisanty O., Rentier-Delrue F., Smal J., Yaron Z., Naor Z. (1995). Hypothalamic and thyroidal regulation of growth hormone in tilapia. Gen. Comp. Endocrinol..

[14] Kagabu Y., Mishiba T., Okino T., Yanagisawa T. (1998). Effects of thyrotropin-releasing hormone and its metabolites, cyclo(His-Pro) and TRH-OH, on growth hormone and prolactin synthesis in primary cultured pituitary cells of the common carp, *Cyprinus carpio*. Gen. Comp. Endocrinol..

[15] Larsen D.A., Swanson P., Dickey J.T., Rivier J., Dickhoff W.W. (1998). In vitro thyrotropin-releasing activity of corticotropin-releasing hormone-family peptides in coho salmon, *Oncorhynchus kisutch*. Gen. Comp. Endocrinol..

[16] Aoki Y., Takahashi M., Masuda T., Tsukamoto T., Iigo M., Yanagisawa T. (2005). Molecular cloning of prepro-thyrotropin-releasing hormone cDNAs from the common carp *Cyprinus carpio* and goldfish *Carassius auratus*. Gen. Comp. Endocrinol..

[17] Iziga R., Ponce M., Infante C., Rebordinos L., Canavate J.P., Manchado M. (2010). Molecular characterization and gene expression of thyrotropin-releasing hormone in Senegalese sole (*Solea senegalensis*). Comp. Biochem. Physiol. B.

[18] Ruiz-Jarabo I., Martos-sitcha J.A., Barragan-Mendez C., Martinez-Rodriguez G., Mancera J.M., Arjona F.J. (2018). Gene expression of thyrotropin- and corticotrophin-releasing hormones is regulated by environmental salinity in the euryhaline teleost Sparus aurata. Fish Physiol. Biochem..

[19] Jiang X.L., Cai Z.W., Zhao X.F., Zhang L.F., Chen Z., Wang Y., Guo X.L., Xu N.Y. (2011). Mapping, cDNA cloning and tissue expression of the porcine thyrotropin-releasing hormone receptor gene. Anim. Biotechnol..

[20] Mekuchi M., Saito Y., Aoki Y., Masuda T., Iigo M., Yanagisawa T. (2011). Molecular cloning, gene structure, molecular evolution and expression analyses of thyrotropin-releasing hormone receptors from medaka (*Oryzias latipes*). Gen. Comp. Endocrinol..

[21] Senthilkumaran B., Sudhakumari C., Mamta S., Raghuveer K., Swapna I., Murugananthkumar R. (2015). “Brain sex differentiation” in teleosts: Emerging concepts with potential biomarkers. Gen. Comp. Endocrinol..

[22] Castaneda-Cortes D.C., Fernandino J.I. (2021). Stress and sex determination in fish: From brain to gonads. Int. J. Dev. Biol..

[23] Fisheries Bureau of Agriculture Ministry of China (2021). China Fishery Statistical Yearbook.

[24] Liu C.K. (1944). Rudimentary hermaphroditism in the symbranchoid eel, *Monopterus albus*. Sinensia.

[25] Jang S.H., Zhou F., Xia L.X., Zhao W., Cheng H.H., Zhou R.J. (2006). Construction of a BAC library and identification of dmrt1 gene of the rice field eel, *Monopterus albus*. Biochem. Bioph. Res. Comumun..

[26] Feng K., Luo H.R., Li Y.M., Chen J., Wang Y.P., Sun Y.H., Zhu Z.Y., Hu W. (2017). High efficient gene targeting in rice field eel *Monopterus albus* by transcription activator-like effector nucleases. Sci. Bull..

[27] Qu X.C., Wang H.P., Piferrer F., Chen S.L., Shen Z.G. (2018). Sex determination and control in eels. Sex Control in Aquaculture.

[28] Hu Q., Guo W., Gao Y., Tang R., Li D.P. (2014). Reference gene selection for real-time RT-PCR normalization in rice field eel (*Monopterus albus*) during gonad development. Fish Physiol. Biochem..

[29] Green M.R., Sambrook J. (2014). Molecular Cloning: A Laboratory Manual.

[30] Kapustin Y., Souvorov A., Tatusova T., Lipman D. (2008). Splign: Algorithms for computing spliced alignments with identification of paralogs. Bio. Direct.

[31] Armenteros J.J., Tsirigos K.D., Sonderby C.K., Petersen T.N., Winther O., Brunak S., von Heijne G., Nielsen H. (2019). SignalP 5.0 improves signal peptide predictions using deep neural networks. Nat. Biotechnol..

[32] Larkin M.A., Blackshields G., Brown N.P., Chenna R., McGettigan P.A., McWilliam H., Valentin F., Wallace I.M., Wilm A., Lopez R. (2007). Clustal W and Clustal X version 2.0. Bioinformatics.

[33] Kumar S., Stecher G., Tamura K. (2016). MEGA7: Molecular evolutionary genetics analysis version 7.0 for bigger datasets. Mol. Biol. Evol..

[34] Livak K.J., Schmittgen T.D. (2001). Analysis of relative gene expression data using real time quantitative PCR and the 2^-^^△△Ct^ method. Methods.

[35] Tse M.C.L., Wong G.K.P., Xiao P., Cheng C.H.K., Chan M. (2008). Down-regulation of goldfish (*Carassius auratus*) prolactin gene expression by dopamine and thyrotropin releasing hormone. Gen. Comp. Endocrinol..

[36] Van Goor F., Goldberg J.I., Wong A.O.L., Jobin R.M., Chang J.P. (1994). Morphological identification of live gonadotropin, growth-hormone, and prolactin cells in goldfish (*Carassius auratus*) pituitary-cell cultures. Cell Tissue Res..

[37] Zhang Y., Zhang S., Liu Z.X., Zhang L.H., Zhang W.M. (2013). Epigenetic modifications during sex change repress gonadotropin stimulation of *cyp19a1a* in a teleost rice field eel (*Monopterus albus*). Endocrinology.

[38] Feng K., Luo H.R., Hou M.X., Li Y.M., Chen J., Zhu Z.Y., Hu W. (2018). Alternative splicing of GnRH2 and GnRH2-associated peptide plays roles in gonadal differentiation of the rice field eel, *Monopterus albus*. Gen. Comp. Endocrinol..

[39] Abbott M., Volkoff H. (2011). Thyrotropin releasing hormone (TRH) in goldfish (*Carassius auratus*): Role in the regulation of feeding and locomotor behaviors and interactions with the orexin system and cocaine- and amphetamine regulated transcript (CART). Horm. Behav..

[40] Vandenborne K., Roelens S.A., Darras V.M., Kuhn E.R., Van der Geyten S. (2005). Cloning and hypothalamic distribution of the chicken thyrotropin-releasing hormone precursor cDNA. J. Endocrinol..

[41] Lechan R.M., Wu P., Jackson I.M.D., Wolf H., Cooperman S., Mandel G., Goodman R.D. (1986). Thyrotropin-releasing hormone precursor: Characterization in rat brain. Science.

[42] Yamada M., Radovick S., Wondisford F.E., Nakayama Y., Weintraub B.D., Wilber J.F. (1990). Cloning and structure of human genomic DNA and hypothalamic cDNA encoding human preprothyrotropin-releasing hormone. Mol. Endocrinol..

[43] Bulant M., Richter K., Kuchler K., Kreil G. (1992). A cDNA from brain of *Xenopus laevis* coding for a new precursor of thyrotropin-releasing hormone. FEBS Lett..

[44] Aoki Y., Masuda T., Iigo M., Yanagisawa T. (2007). Molecular cloning of prepro-thyrotropin-releasing hormone cDNA from medaka (*Oryzias latipes*). Gen. Comp. Endocrinol..

[45] Kuchler K., Richter K., Trnovsky J., Egger R., Kreil G. (1990). Two precursors of thyrotropin-releasing hormone from skin of *Xenopus laevis*. J. Biol. Chem..

[46] Singh O., Pradhan D.R., Nagalakashmi B., Kumar S., Mitra S., Sagarkar S., Sakharkar A.J., Lechan R.M., Singru P.S. (2019). Thyrotropin-releasing hormone (TRH) in the brain and pituitary of the teleost, *Clarias batrachus* and its role in regulation of hypophysiotropic dopamine neurons. J. Comp. Neurol..

[47] Ojima D., Iwata M. (2010). Central administration of growth hormone-releasing hormone and corticotropin-releasing hormone stimulate downstream movement and thyroxine secretion in fall-smolting coho salmon (*Oncorhynchus kisutch*). Gen. Comp. Endocrinol..

[48] Bromage N.R. (1975). The effects of mammalian thyrotropin-releasing hormone on the pituitary-thyroid axis of teleost fish. Gen. Comp. Endocrinol..

[49] Matz S.P., Hofeldt G.T. (1999). Immunohistochemical localization of corticotropin-releasing factor in the brain and corticotropin-releasing factor and thyrotropin-stimulating hormone in the pituitary of chinook salmon (*Oncorhynchus tshawytscha*). Gen. Comp. Endocrinol..

[50] Tovo-Neto A., Rodrigues M.S., Habibi H.R., Nobrega R.H. (2018). Thyroid hormone actions on male reproductive system of teleost fish. Gen. Comp. Endocrinol..

[51] Kaneko H., Ijiri S., Kobayashi T., Izumi H., Kuramochi Y., Wang D.S., Mizuno S., Nagahama Y. (2015). Gonadal soma-derived factor (*gsdf*), a TGF-beta superfamily gene, induces testis differentiation in the teleost fish *Oreochromis niloticus*. Mol. Cell. Endocrinol..

[52] Pfennig F., Standke A., Gutzeit H.O. (2015). The role of Amh signaling in teleost fish—Multiple functions not restricted to the gonads. Gen. Comp. Endocrinol..

[53] Guiguen Y., Fostier A., Piferrer F., Chang C.F. (2010). Ovarian aromatase and estrogens: A pivotal role for gonadal sex differentiation and sex change in fish. Gen. Comp. Endocrinol..

[54] Hu Q., Guo W., Gao Y., Tang R., Li D.P. (2015). Molecular cloning and characterization of *amh* and *dax1* genes and their expression during sex inversion in rice field eel *Monopterus albus*. Sci. Rep..

[55] Zhu Y.F., Wang C.L., Chen X.W., Guan G.J. (2016). Identification of gonadal soma-derived factor involvement in *Monopterus albus* (protogynous rice field eel) sex change. Mol. Biol. Rep..

[56] Zhang Y., Zhang W.M., Yang H.Y., Zhou W.L., Hu C.Q., Zhang L.H. (2008). Two cytochrome P450 aromatase genes in the hermaphrodite rice field eel *Monopterus albus*: mRNA expression during ovarian development and sex change. J. Endocrinol..

[57] Yang Y., Liu Q.H., Xiao Y.H., Xu S.H., Wang X.Y., Yang J.K., Song Z.C., You F., Li J. (2020). Effects of environmental stress (sex steroids and heat) during sex differentiation in Japanese flounder (*Paralichthys olivaceus*): Insight from germ cell proliferation and *gsdf-amh-cyp19a1a* expression. Aquaculture.

