# Supplementary material for: Molecular Cloning and Expression Analysis of Thyrotropin-Releasing Hormone, and Its Possible Role in Gonadal Differentiation in Rice Field eel Monopterus albus
Source: Animals (Basel). 2022 Jun 30;12(13):1691. doi: 10.3390/ani12131691 (PMC9264984; doi:10.3390/ani12131691)
Supplement: Supplementary file 1 [file animals-12-01691-s001.zip › animals-1703846-supplementary.pdf]

Table S1. The relative expression data of *trh* in various tissues

Tissues	Relative expression of *trh*
heart	2.2 ± 0.6[a]
liver	1.3 ± 0.2[a]
spleen	2.9 ± 0.7[a]
kindey	1.3 ± 0.3[a]
intestin	1.0 ± 0.3[a]
muscle	40.4 ± 4.1[c]
telencephalon	25153.2 ± 6974.4[f]
mesencephalon	10572.7 ± 2222.8[e]
cerebellum	14674.6 ± 2129.7[ef]
medulla oblongata	11128.9 ± 889.8[e]
hypothalamus	17951.7 ± 3543.4[ef]
pituitary	12.4 ± 5.6[b]
ovary	168.1 ± 5.5[d]
testis	179.6 ± 77.6[d]

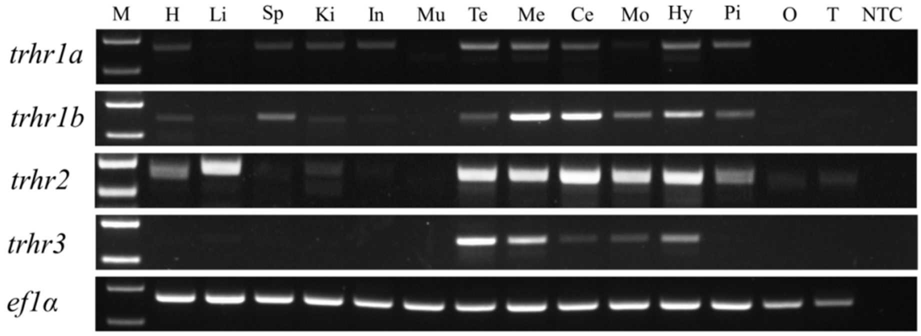

Figure S1. Distribution of *trhr* in rice field eel tissues

M: marker; H: heart; Li: liver; Sp: spleen; Ki: kidney; In: intestine; Mu: muscle; Te: telencephalon; Me: mesencephalon; Ce: cerebellum; Mo: medulla oblongata; Hy: hypothalamus; Pi: pituitary; O: ovary; T: testis; NTC: no template control.

Table S2. Primers used for the detection of tissue distribution of *trhr* by RT-PCR.

Name of primer	Sequence of primer (5'→3')	Product size (bp)
trhr1a-F	AAAATCATCATGCTCGTCTG	408
trhr1a-R	GTAGGGCATCCAAAGTAAGG	
trhr1b-F	ATTTCGGCGTCTTCTTCGTG	375
trhr1b-R	CGTTGTAGATGACCGGGTTG	
trhr2-F	ATAGCGATCTGCCATCCAAT	423
trhr2-R	TGCCAGCATCTTAGTCACCT	
trhr3-F	GCCCTCAAATCTGAATGACC	365
trhr3-F	AGAACATCGGCACATCGTAC	
ef1a-F	AGCGTGGTATCACCATTGAC	407
ef1a-R	CCTCCAGCATGTTGTCTCC	
